# A Survey of U.S Adults’ Opinions about Conduct of a Nationwide Precision Medicine Initiative® Cohort Study of Genes and Environment

**DOI:** 10.1371/journal.pone.0160461

**Published:** 2016-08-17

**Authors:** David J. Kaufman, Rebecca Baker, Lauren C. Milner, Stephanie Devaney, Kathy L. Hudson

**Affiliations:** 1 National Human Genome Research Institute, Division of Genomics and Society, National Institutes of Health, Rockville, MD, United States of America; 2 National Institutes of Health, Office of the Director, Bethesda, MD, United States of America; Universidad Veracruzana, MEXICO

## Abstract

**Objectives:**

A survey of a population-based sample of U.S adults was conducted to measure their attitudes about, and inform the design of the Precision Medicine Initiative’s planned national cohort study.

**Methods:**

An online survey was conducted by GfK between May and June of 2015. The influence of different consent models on willingness to share data was examined by randomizing participants to one of eight consent scenarios.

**Results:**

Of 4,777 people invited to take the survey, 2,706 responded and 2,601 (54% response rate) provided valid responses. Most respondents (79%) supported the proposed study, and 54% said they would definitely or probably participate if asked. Support for and willingness to participate in the study varied little among demographic groups; younger respondents, LGBT respondents, and those with more years of education were significantly more likely to take part if asked. The most important study incentive that the survey asked about was learning about one’s own health information. Willingness to share data and samples under broad, study-by-study, menu and dynamic consent models was similar when a statement about transparency was included in the consent scenarios. Respondents were generally interested in taking part in several governance functions of the cohort study.

**Conclusions:**

A large majority of the U.S. adults who responded to the survey supported a large national cohort study. Levels of support for the study and willingness to participate were both consistent across most demographic groups. The opportunity to learn health information about one’s self from the study appears to be a strong motivation to participate.

## Introduction

Precision medicine is an emerging approach to disease prevention, diagnosis and treatment that takes into account differences between individuals. While not new, to date it has only been applied to certain conditions. The Precision Medicine Initiative® (PMI) plans to build a comprehensive scientific knowledge base to implement precision medicine on a larger scale by launching a national cohort study of a million or more Americans [1]. The national cohort study will aim to foster open, responsible data sharing, maintain participant privacy, and build on strong partnerships between researchers and participants [2].

Prospective cohort studies using biospecimens are a common approach taken to examine the effects and interactions of genes, environment, and lifestyle [3–7]. Although they are labor-, time-, and capital-intensive [8], these studies can provide the statistical power needed to detect small biological effects on disease [9–10]. Both public [3,5,11,12] and private [4,6,7] cohort studies and biobanks have been created, and genetic analyses have been incorporated into existing cohort studies as genotyping and computational tools become more accessible [11,13,14].

The Precision Medicine Initiative® aims to expand on these efforts to engage a wider group who would volunteer a standardized set of health information that can be shared broadly with qualified researchers. Cohort volunteers would share their information and biological specimens for genomic and other analyses. Genomic information would be combined with clinical data from electronic health records, lifestyle data, and data measured through mobile health devices, for use by a broad range of researchers. Participants would have access to information about cohort-fueled research findings, as well as some individual research results.

The National Institutes of Health (NIH) along with other federal agencies has begun to design and execute this large prospective study as part of the White House’s Precision Medicine Initiative® [1,2,10]. During the initial planning process for this PMI Cohort Program, NIH engaged a wide variety of expertise through four public workshops on issues of design and vision for the cohort [15–18], and two Requests for Information [19, 20].

At a July, 2015 workshop on participant engagement and health equity a broad range of experts discussed the role of participant engagement in the design and conduct of an inclusive PMI cohort [17]. The discussions, which focused on building and sustaining public trust, actively engaging participants, and enlisting participants to set research priorities and directly collect study data informed the strategic design of the PMI cohort [21].

The workshop concluded that continued engagement of a broad range of stakeholders will be needed to plan, carry out and sustain the PMI cohort program. As part of this larger public engagement effort, a survey of U. S. adults was conducted to measure support for such a study, to measure acceptability of various design features, and to identify and prioritize public concerns.

## Materials and Methods

### Survey Methods

A 44-question online survey, determined by the NIH Office of Human Subjects Research to be exempt from human subjects review (Exemption #12889), collected U.S. adults’ opinions about a national cohort study. Formal consent was not obtained both because the study was judged as exempt, and because completion of the survey is taken as a form of consent to participate.

The survey was not intended to collect psychometric data and thus did not rely on validated psychometric scales. However, to examine changes over time in public support for and hypothetical willingness to take part in a large U.S. cohort study, exact wording from two previous surveys was used for some questions.[22–24] Some questions came from a related survey of biobank participants under development at the time by the NIH-funded Electronic Medical Records and Genomics (or eMERGE) consortia [25]. Response choices consisted of pre-defined options. Most of these question choices were developed based on findings of focus groups conducted as part of prior studies [22–24].

The survey addressed support for and willingness to take part in the cohort study, specific aspects of participation, study oversight including participant involvement in governance, and the return of information to participants. Respondents were first shown a description of the cohort study. (S1 Appendix) At the end of the description, respondents were told that participants in the cohort study “might get access to the information collected about their health”.

Respondents were then asked several questions about their support for the concept and willingness to take part if they were asked. (S2 Appendix contains exact wording of all of the questions analyzed here.) Respondents were also shown one of eight different scenarios, selected at random, describing study consent and data sharing and asked whether they would “consent to share your samples and information with researchers in this manner”. The eight scenarios varied with respect to two factors: the structure of consent (broad, study by study, menu, or dynamic consent) and the presence or absence of a statement that cohort study participants would “have access to a website where you would be able to see what studies are going on, which studies are using your information, and what each study has learned.” The exact wording of all eight versions of consent is found in S3 Appendix.

A pilot survey (n = 30) fielded between May 5 and May 7, 2015 evaluated the instrument length and logic. Median completion time for the pilot was 23 minutes; the instrument was shortened to 20 minutes. The final instrument was translated into Spanish for use by respondents who preferred it. The translation was back-translated and certified.

Sample selection and online survey administration was managed by the online survey firm GfK. The survey sample was drawn from GfK’s KnowledgePanel, which is itself a probability-based pool of approximately 55,000 people designed to be representative of the U.S. population.

Individuals can become GfK panelists only after being randomly selected by GfK; no one can volunteer to be a member. GfK selects people using probability-based sampling of addresses from the U.S. Postal Service’s Delivery Sequence File, which includes 97% of residential U.S. households. Excluded from eligibility are those in residential care settings, institutionalized and incarcerated people, and homeless individuals. Individuals residing at randomly sampled addresses are invited to join KnowledgePanel through a series of mailings in English and Spanish; non-responders are phoned when a telephone number can be matched to the sampled address.

For those who agree to be part of the GfK panel, but do not have Internet access, GfK provides at no cost a laptop and Internet connection. GfK also optimized the survey for administration via smartphone or tablet. When GfK enrolls participants into its panel, each panel participant answers demographic questions which are banked and periodically updated by GfK. GfK can then provide data on common demographics for each of its participants, allowing surveys to reduce burden by not asking these questions. Data in this paper on participants’ self-reported race and ethnic group, age, education, gender, sexual orientation or gender identity, household income, and residence in a metropolitan statistical area were all measured by GfK prior to this survey.

GfK attempts to address sources of survey error including sampling error, non-coverage and non-response due to panel recruitment methods and panel attrition by using demographic post-stratification weights based on demographics of the U.S. Current Population Survey (CPS) as the benchmark. Once the data are collected, post-stratification weights are constructed so that the study data can be adjusted for the study’s sample design and for survey nonresponse[26].

This series of methods has resulted in GfK survey samples that compare favorably to other gold standard methods designed to generate population-based samples [27]. During the field period for this survey, GfK first drew a random sample of 3,271 U.S. adults from their Web-enabled panel of approximately 55,000 U.S. residents. This included Hispanics and black non-Hispanics. In order to meet oversampling goals of 500 in these two groups, three additional random samples were drawn, including one of 665 Black non-Hispanic adults and two additional samples of 541 and 320 Hispanic adults. GfK contacted each of these 4,777 individuals via email to invite them to take part in this survey. Non-respondents received up to four email reminders from GfK.

The survey was fielded online between May 28, 2015 and June 9, 2015. Participants received the equivalent of $2 for their time. After survey data were collected, information previously collected by GfK on panel members’ demographics was added to the dataset.

### Analysis Methods

Data were cleaned and analyzed using SPSS software [28]. Respondents who skipped more than one-third of the questions, or who completed the survey in less than one quarter of the median survey time were excluded from analyses.

Support for the study and willingness to participate were measured using 4-point Likert scales; two binary variables were created for analysis from these scales. Demographic variables were analyzed using the categories as shown in Table 1. Two sets of multiple logistic regressions were conducted. (Table 2) Support for the study and willingness to participate were the dependent variables. In these models, race and ethnic group were treated as a single categorical variable using dummy variables, and treating white non-Hispanics as the reference group. Education, household income and age were each modeled as ordinal variables using the categories shown in Tables 1 and 2. Respondents who identified as lesbian, gay, bisexual or transgender (LGBT) were analyzed together as a single group.

**Table 1:** Weighted and unweighted demographics of survey respondents, compared to 2010 U.S. Census figures. (n = 2,601).

	Demographic Group	Unweighted N	Unweighted %	Weighted N	Weighted %	2010 U.S. Census (over 18)
*Total*		2,601	100%	2,601	100%	
*Gender*	Men	1,252	48%	1,251	48%	49%
* *	Women	1,349	52%	1,350	52%	51%
*Race and Ethnic Group*	White, non-Hispanic	1,421	55%	1,721	66%	68%
	Black, non-Hispanic	505	19%	295	11%	12%
* *	Hispanic (all races)	523	20%	385	15%	14%
* *	Other non-Hispanic	152	6%	200	8%	6%
*Survey language*, *among Hispanics*	Spanish	255	10%	186	7%	NA
	English	268	10%	199	8%	NA
*Age*	21–29	350	14%	447	17%	18%
* *	30–44	599	23%	694	27%	28%
* *	45–59	798	32%	736	28%	29%
* *	60+	854	33%	724	28%	25%
*Household Income*	<$30,000	616	24%	616	24%	24%
	$30,000–$59,999	657	25%	657	25%	26%
* *	$60,000–$99,999	624	24%	624	24%	23%
* *	$100,000+	704	27%	704	27%	25%
*Education*	0–11 Years	273	10%	297	11%	19%
* *	High School	723	28%	773	30%	27%
* *	Some College	778	30%	731	28%	27%
* *	B.A.	827	32%	800	31%	28%
*Residence in Metropolitan Statistical Area*	Metro	2258	87%	2195	84%	81%
	Non-Metro	343	13%	406	16%	19%
*Lesbian*, *Gay*, *Bisexual or Transgender*		212	8%	203	8%	~4%

**Table 2:** Results of two multiple logistic regressions examining demographic factors related to survey respondents’ support for the national cohort study, and their willingness to participate in the study if asked (n = 2,601). Each multiple logistic regression included independent covariates for gender, self-identified race and ethnic group, survey language (among Hispanics only), age, household income, educational attainment, residence within or outside a metropolitan statistical area, and identification as either lesbian, gay, bisexual or transgender. For purposes of the analysis, race and ethnicity was treated as a categorical variable, using dummy variables for black non-Hispanics, Hispanics, and other non-white, non-Hispanics. Education, household income, and age were each treated as 4-level variables using the categories shown below. To examine whether there were differences among Hispanics who took the survey in Spanish and English, separate regressions were conducted using Hispanic respondents’ data only, adjusting for all of the variables below except for race and ethnic group.

	Demographic Group	Weighted N	% who said the study definitely or probably should be done	Beta	S.E.	p-value	% definitely or probably willing to participate in hypothetical biobank	Beta	S.E.	p-value
*Total*		2,601	79%				54%			
*Gender*	Men	1,251	77%	-0.160	0.098	0.10	54%	0.007	0.080	0.93
* *	Women	1,350	80%	ref			54%	ref		
*Race and Ethnic Group*	White, non-Hispanic	1,721	79%	ref			53%	ref		
	Black, non-Hispanic	296	77%	-0.032	0.156	0.84	55%	0.100	0.131	0.44
* *	Hispanic (all races)	385	78%	0.136	0.149	0.36	59%	0.291	0.123	0.02
* *	Other non-Hispanic	200	81%	0.555	0.541	0.31	56%	0.058	0.167	0.73
*Survey language*, *among Hispanics*	Spanish	186	80%	0.159	0.252	0.53	61%	0.383	0.205	0.06
	English	199	80%	ref			56%	ref		
*Age*	21–29	447	81%	-0.047	0.048	0.32	60%	-0.156	0.039	<0.0001
* *	30–44	694	80%				58%			
* *	45–59	735	79%				53%			
* *	60+	724	77%				47%			
*Household Income*	<$30,000	616	73%	0.106	0.050	0.04	55%	-0.040	0.041	0.96
	$30,000–$59,999	657	78%				52%			
* *	$60,000–$99,999	624	81%				52%			
* *	$100,000+	704	85%				57%			
*Education*	0–11 Years	297	69%	0.302	0.057	<0.0001	49%	0.230	0.047	<0.0001
* *	High School	773	74%				48%			
* *	Some College	731	80%				55%			
* *	B.A.	800	87%				60%			
*Residence in Metropolitan Statistical Area*	Metro	2195	80%	ref			55%	ref		
	Non-Metro	406	73%	-0.281	0.130	0.03	50%	-0.06	0.113	0.62
*Lesbian*, *Gay*, *Bisexual or Transgender*		203	82%	0.197	0.191	0.30	64%	0.390	0.155	0.01

The multiple logistic regressions examined demographic factors associated with support and participation. Analyses that included the entire sample were weighted to 2014 U.S. Census demographic benchmarks. To examine whether Hispanics who took the survey in English differed from those who took it in Spanish with respect to support and willingness to participate, separate regressions were carried out using only Hispanic respondents’ data, adjusting for the covariates in Table 1 except for race and ethnic group. Analyses within or between different races and ethnic groups used an alternate set of weights calculated for these oversampled groups.

## Results

In total, 4,777 people were contacted by GfK via email and invited to take the survey, and 2,706 provided at least some responses, for an overall response rate of 56.6%. Overall response rates were 62.2% (2,036 of 3,271) in the general population sample, 345/665 or 51.9%, in the Black-non-Hispanic oversample and 325/841 or 38.6% in the Hispanic oversample. It should be noted that 320 Hispanic cases in the oversample were invited to respond on June 5, 2015 and the survey was closed on June 9, 2015. Members of this oversample received fewer email reminders to take part and had a shorter field period to participate, which could account for some but not all of the lower completion rate in the Hispanic oversample.

Responses from 105 people (3.9%) were excluded from analysis because they skipped more than one-third of the questions or completed the survey in less than 6 minutes, leaving a valid response rate of 2,601/4,777 or 54%. The excluded people did not differ demographically from those retained in the analysis. The margin of error on opinion estimates based on the sample of 2,601 is +/- 1.9%.

Demographic characteristics of the surveyed population are found in Table 1. After weighting the sample, people with less than twelve years of education were still somewhat underrepresented compared to census data. This should be considered where differences in opinions exist across education groups.

### General Support for the Cohort Study

Immediately after viewing the description of the cohort study, participants were asked ‘Based on the description you just read, do you think this study should be done?’ Seventy-nine percent said the study definitely (22%) or probably (57%) should be done, while 21% said probably not (16%) or definitely not (5%).

Similar levels of support were observed across most demographic groups (Table 2). A multiple logistic regression treating support for the cohort study as a binary independent variable showed that, adjusting for the other factors in Table 1, no significant differences in support were observed between genders, age groups, races or ethnic groups, or between Hispanics who took the survey in Spanish and English. Fewer years of education (p<0.0001), lower household income (p = 0.04), and residence outside of metropolitan statistical areas (a proxy for rural residence, p = 0.03) were independently associated with lower levels of support for the study. However, in all but one of the demographic categories examined (0–11 years of education), 70% or more said they supported the study.

### Stated Willingness to Participate in the Cohort Study if Asked

The question ‘Would you participate in the cohort study if you were asked?’ was also posed at the survey’s start. Prior to this question, the only possible personal benefit of participating that was mentioned was that cohort participants “might get access to the information collected about their health.” A majority of participants (54%) said they definitely (14%) or probably (40%) would participate if asked, while the rest said they probably (30%) or definitely (16%) would not take part. Willingness to participate did not vary considerably between demographic groups (Table 2). Majorities (>50%) in most groups said they would participate if asked, and in each group, at least 1 in 9 people said they would definitely take part. A second multiple logistic regression treated willingness to participate as a binary independent variable. Adjusting for the other factors in Table 1, increasing years of education (p<0.0001) and younger age (p<0.0001) were independently associated with increased likelihood of willingness to participate. Compared to white non-Hispanics, Hispanic respondents were more likely to say they would participate (59% vs 53%, adjusted p = 0.009). As a group, respondents who identified as lesbian, gay, bisexual or transgender were significantly more likely to say they would participate if asked (p = 0.01).

One in four respondents said that they supported the idea of the study, but also said they would not participate if they were asked. People who supported the study but would not participate if asked were more than twice as likely as those who would participate to agree that the study “would take too much of my time” (77% vs 30%), and were less likely than those who would take part to agree with the statement “I trust the study to protect my privacy” (51% vs. 81% respectively).

The survey was not designed to educate people about precision medicine or biomedical research. However it was hypothesized that thinking about some of the attitudinal questions in the survey could influence respondents’ opinions about taking part in the study. To test this hypothesis, near the end of the survey, respondents were asked again “Now that you have had a chance to think about the study, would you participate in the study if you were asked?” Overall, responses were fairly similar to the earlier question– 56% said they would definitely (15%) or probably (41%) take part if asked; 25% said they probably would not participate and 19% said they definitely would not. Seven in ten (70%) did not change their answer from the beginning to the end of the survey. However, 15% had grown more positive about participating by the end of the survey, and 15% had grown more negative. Some demographic differences were observed in shifts from the beginning of the survey to the end. For example, fewer people who took the survey in Spanish (61% vs 55%) said they would take part at the end of the survey, while more people with some college (55% vs 60%) or a bachelor’s degree (60% vs 64%) were willing to take part at the end of the survey.

Respondents were also asked about specific things they would be willing to do as study participants. Among all respondents, one in seven (14%) said they would participate for their lifetime, and an additional 11% said they would take part for at least ten years. Among people who said they would definitely or probably participate if asked, 42% said they would take part for at least ten years. However, only one in four of the Black non-Hispanics and Hispanics who were willing to take part said they would do so for at least ten years.

All respondents, including those who said they would not participate, were asked to “[i]magine you were considering participating in the study”, and then asked about their willingness to provide various types of data and samples. Nearly three quarters of respondents (73%) said that if they were participating they would be willing to provide the study with a blood sample. Higher fractions said they would provide urine, hair, and saliva samples (75%), data from an activity tracker (i.e. Fitbit) (75%), genetic information (76%), a family medical history (77%), soil and water samples from home (83%), and data on lifestyle, diet and exercise (84%). Among those with a social media account (n = 1,641) only 43% responded that they would share their social media information with the study.

In each demographic group listed in Table 1, at least 9% of people (one in eleven) said they would definitely participate, would take part for at least ten years, and would provide the study with a blood sample.

In the sample, 87% owned either a smart phone (62%) or a cell phone (25%). Three-quarters of these phone users responded that if they “were texted or prompted on your cell phone to answer a question from the study, or measure your pulse”, they would be willing to respond at least once a week. A majority (59%) said they would respond at least once a day, and 28% would be willing to respond at least twice a day.

### Incentives for participation

Respondents were asked about the importance of six different incentives in their decision about whether or not to participate. The most important incentive was “learning information about my health”, listed as either somewhat or very important by 90% of people, including at least 85% of people in each group in Table 1. Receiving payment for their time (80%) and getting health care (77%) were important to more people than receiving free internet connections (56%), activity trackers (55%) or smartphones and data plans (52%). However, the technology incentives were of more interest to younger respondents, those with lower household incomes, and those with fewer years of education.

**Fig 1:**
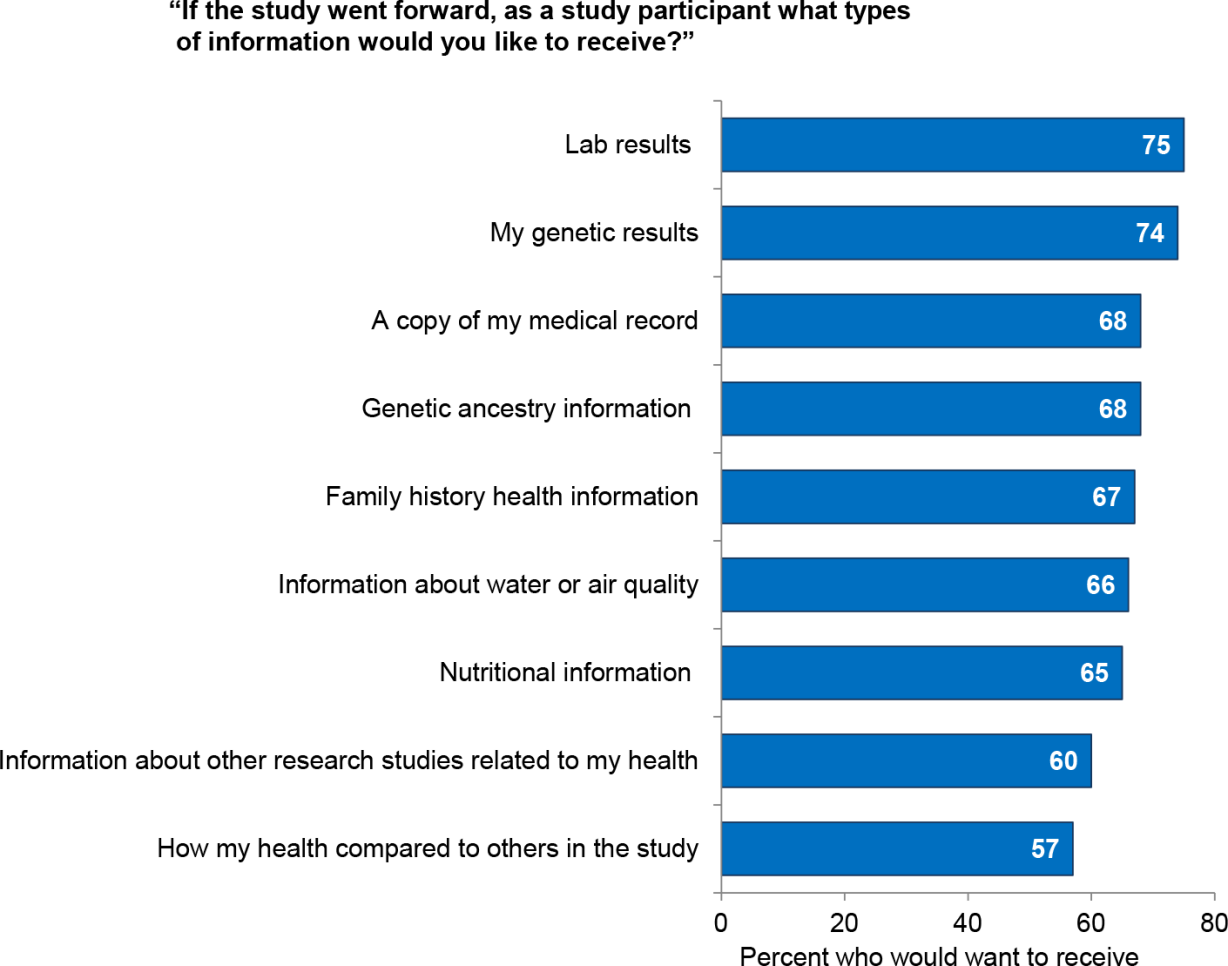
Results of a survey of 2,601 U.S. adults about participation in a nationwide longitudinal study of genes and environment. Respondents’ interest in different types of information the study could return to participants.

Respondents said they would be interested in a wide variety of information that the study might return to them (Fig 1). Three in four would be interested in “lab results” (examples given were cholesterol and blood sugar) as well as genetic results. Slightly fewer (68%) said they would like a copy of their medical record. Six in ten (60%) said they would be interested in receiving information about other research studies related to their health.

### Consent and Sharing of Data and Samples

As described above, respondents were randomly selected to view one of eight consent scenarios and asked “would you consent to share your samples and information with researchers in this manner”. There were four models of consent: broad, study-by-study, menu, and dynamic consent. The exact wording of the four consent scenarios is found in S3 Appendix. Two versions of these four scenarios were presented; four where the consent description stood alone, and four where the consent option was followed by this sentence: “You would (also) have access to a website where you would be able to see what studies are going on, which studies are using your information, and what each study has learned.”

**Fig 2:**
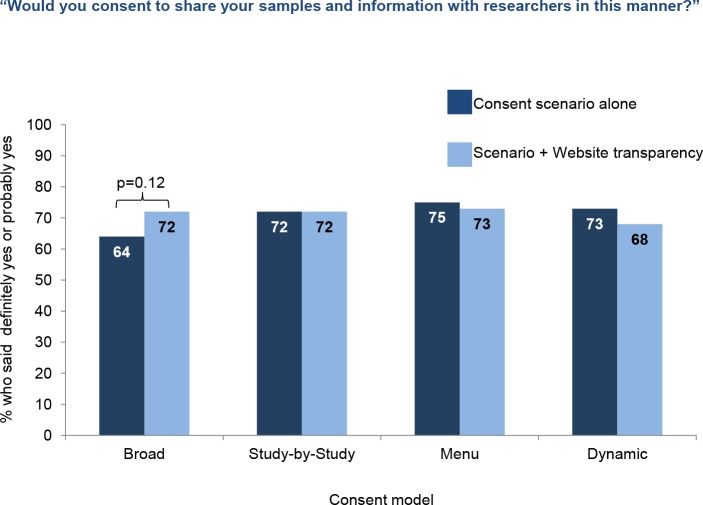
Results of a survey of 2,601 U.S. adults about participation in a nationwide longitudinal study of genes and environment. Willingness to share samples and information under different consent models.

When the consent models were displayed alone, similar fractions of respondents said they would share samples and data under the study-by-study (72%), menu (75%), and dynamic consent models (73%) (Fig 2) while 64% would share with the study under the broad consent model. However, when the consent scenarios were accompanied by the statement about a website that displays how samples and data are being used, there was essentially no difference in support for the four consent models.

When asked about allowing different categories of researchers to use their samples and information, people were most likely to say they would share with researchers at the NIH (79%) and U.S. academic researchers (71%). There was more reluctance to share with “pharmaceutical or drug company researchers” (52%) or “other government researchers” (44%). The category “other government researchers” may be overly broad and non-specific; for example, had the survey named researchers at specific health-related agencies, responses may have differed. Consistent with two prior surveys, people were least willing to share with university researchers in other countries (39%) [23, 24].

In a separate question, 43% of people agreed that if their personal information was removed first, they would be willing to have their “information and research results available on the Internet to anyone”.

### Involvement of Participants in Design and Conduct of the Study

To create a cohort study that addresses health related questions that are relevant to the lives of participants, study designers are embracing new models of participants as partners in research. Several questions addressed respondents’ interest in this area. A large majority (76%) agreed with the statement “research participants and researchers should be equal partners in the study”.

**Fig 3:**
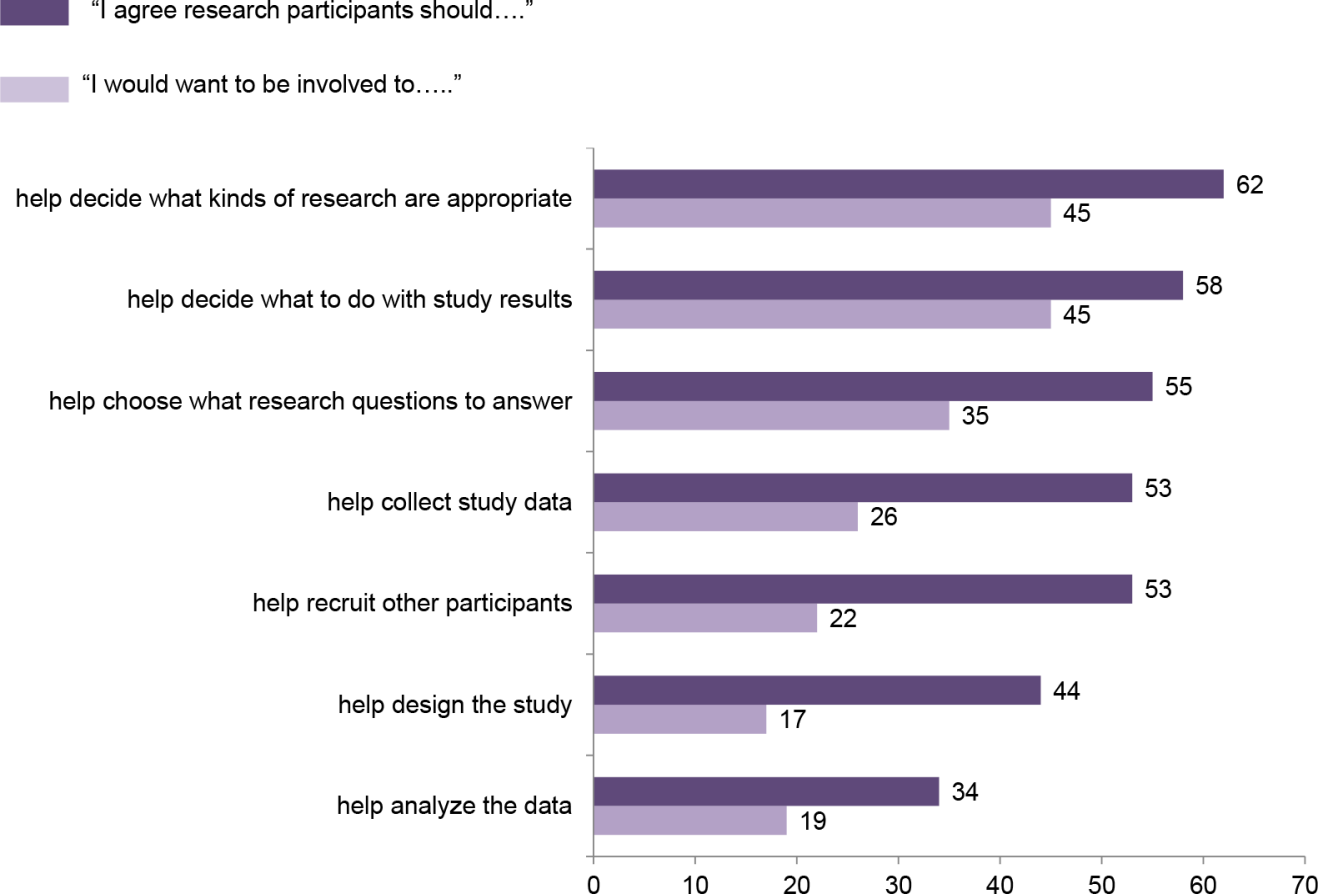
Results of a survey of 2,601 U.S. adults about participation in a nationwide longitudinal study of genes and environment. Aspects of the study that participants should be involved in generally, and aspects the respondent themselves would want to be involved in.

Fig 3 shows that between 34% and 62% of respondents said that participants should be involved in various phases of the study. Important to the most respondents was participant involvement in three governance tasks—deciding what kinds of research are appropriate, deciding what to do with study results, and deciding what research questions to answer. Between 35% and 45% said they would like to be involved themselves in those three aspects of the study.

One in four people said that including research participants in planning and running the study would increase their willingness to participate, including 18% of those people who said earlier in the survey that they would not take part if asked. Another 17% said it would make them less willing to take part if participants were included, and 58% said it would not affect their decision.

## Discussion

The Precision Medicine Initiative® cohort program been engaging and partnering with participant representatives prior to the launch of the study, and plans to actively continue this work with cohort study participants. This survey reflects an early effort to understand the views and preferences of potential participants toward the PMI cohort program. Findings from this survey were incorporated into the final report that the Precision Medicine Initiative Working Group made to the National Institutes of Health, and are reflected in recommendations about how the cohort study might be designed.[21] As such, the survey represents one of several early efforts to engage the public in order to inform whether and how the PMI cohort study might move forward.

Across most demographic groups this survey found consistent levels of support for and willingness to participate in the PMI cohort study as it was described. The overall support (79%) and willingness to take part (54%) observed are comparable to measures in previous public surveys conducted using nationwide GfK samples in 2008 and 2012, which found overall support for a large nationwide biobank at 84% and 83% respectively, and willingness to participate if asked at 60% and 56% respectively [23, 25]. Support in this study could be lower than that measured in earlier surveys in part due to the explicitly stated association with the NIH (and thus the federal government) in this survey, as well as high profile privacy breaches associated with the federal government and health providers in the six months prior to the survey [29, 30]. Differences in levels of support and willingness to participate might also result from this survey’s mention of smartphones and activity trackers to collect data, especially among older respondents, who had more concern about the privacy of electronic media (data not shown). This study’s estimates of overall support and willingness to participate are also biased slightly upward, since people with fewer years of education, who were underrepresented in the sample compared to U.S. demographics, were less likely to support the study and participate. However, extrapolating support and willingness observed in each category of education to U.S. census frequencies of the education categories suggests the magnitude of inflation from this source is less than 1% in both figures.

The findings suggest that certain groups including older Americans and those with lower socioeconomic status may require additional engagement if they are to take part. However the survey findings do not support the idea that people from communities that have historically been understudied in research are not interested in participating in this cohort. In contrast, in each demographic group in Table 1, at least one in eleven people (9%) said they would definitely participate if asked, would donate blood, and would take part for at least ten years. The willingness to take part observed here is only the foundation for efforts needed to engage, recruit and retain people in traditionally underrepresented groups. Researchers likely must work as part of communities that have been underrepresented, if those communities are going to feel and be a part of the study. [17] To this end, scientists may consider adopting language and policies that bond researchers and potential participants together todesign and govern the study [17, 31].

Thirty percent of survey respondents shifted their opinions about their willingness to take part in the study from the beginning to the end of the survey. This suggests that considering some of the potential risks and benefits of participation may inform and influence people’s decision to take part. Engagement before and during study recruitment may help people make better informed decisions about participation.

The observation that receipt of health information was the most important incentive was consistent with results of a 2008 nationwide survey [23]. Maximizing information shared with research participants will be a key challenge of the PMI. Survey respondents expressed interest in a wide range of information, including but not limited to genetic information. Laboratory measurements such as blood sugar were seen as equally interesting. The return of information may also benefit research, encouraging participants to stay engaged and enrolled, and to take part in other research studies based on their results.

There was considerable enthusiasm among respondents about participant involvement in different phases of the study. Between 19% and 45% said they themselves would take part in various study related functions. The tasks of greatest interest to the most people were governance-related. Developing “participants as equal partners” may not drastically improve enrollment. However it may establish the kind of study identity and enthusiasm that others have cited as one key to the success of this effort [16–18, 21].

NIH researchers were found to be trusted with the data and samples to be collected. If the NIH serves as a leader in the PMI cohort, it must be prepared to understand and meet those expectations. For example, if PMI cohort data are shared with foreign academics, study leaders may need to address negative attitudes about such sharing, perhaps by engaging the public to understand their reservations, and explaining how the sharing benefits U.S. medicine and research.

### Limitations

It is very important to note that the results of this survey were not meant to, and do not accurately predict what portion of American adults would take part in the PMI cohort study if they were asked. First, although half of respondents said they would take part in PMI if asked, only 54% of the sample contacted for this survey agreed to participate. Second, respondents in this survey are members of the GfK panel; they may be more favorably inclined toward research participation than the general population. This limitation is inherent in most studies of attitudes about taking part in biomedical research, since people must be willing to take part in a survey study like this one to collect such data. On the other hand, the bias may not be particularly strong since sharing opinions on a survey is likely to be a smaller, lower risk commitment than sharing ones’ biospecimens and medical data. Third, people’s stated willingness to take part in a hypothetical study will not correlate perfectly with actual behaviors. The PMI study should not be expected to enjoy a 54% success rate in its recruitment based on these data. Given the limitations of the survey, the data likely provide valid estimates of support for the study as well as the relative willingness of different groups to participate, the relative importance of different incentives, and the relative acceptability of different consent models.

## Supporting Information

S1 Appendix Text used to describe the PMI cohort study in the survey. (DOCX)

S2 Appendix Exact wording of survey questions used in this manuscript. (DOCX)

S3 Appendix Wording used to describe eight consent scenarios in the survey. (DOCX)
