# Supplementary material for: A Survey of U.S Adults’ Opinions about Conduct of a Nationwide Precision Medicine Initiative® Cohort Study of Genes and Environment
Source: PLoS One. 2016 Aug 17;11(8):e0160461. doi: 10.1371/journal.pone.0160461 (PMC4988644; doi:10.1371/journal.pone.0160461)
Supplement: S1 Appendix — (DOCX) [file pone.0160461.s001.docx]

**Supporting Information**

**S1 Appendix**: **Text used to describe the PMI cohort study in the survey.**

Many diseases result from a mix of genetic, environment, and lifestyle factors. To figure out how these factors cause disease, a large number of people need to be studied for a long time. The National Institutes of Health (NIH) is thinking of doing a study of one million volunteers for 10 years or longer. The proposed study would help doctors choose medicines and treatment plans based on a person’s genes, environment, and lifestyle.

Before deciding whether to do the study or not, the NIH wants to know what the public thinks about the idea.

Please read the description of the proposed study below:

The study would collect samples (like blood and saliva) and data from one million volunteers. The study would need to include men and women of all ages, as well people from all places, backgrounds, and cultures found in the United States. The study could also include children.

Before a person decided to take part, someone from the study would explain the risks and benefits of participating. All participants would give their informed consent to be in the study.

Researchers would follow people’s health over time. Participants would give the study access to all of their medical records, as well as information about their diet and lifestyle. They would give a blood sample and other samples for genetic tests and other lab tests like cholesterol and blood sugar. People might be asked to do other things like answer surveys, keep a food diary, or track their daily exercise or heart rate with a device like a Fitbit or a smartphone. People could also be asked for samples from their home, like water or soil.

The samples, information, and lab test results would all be “coded”. “Coded” means that the names and personal information of each participant would be replaced with a number. All coded information would be stored in a secure databank.

Qualified researchers could use the coded samples and data to study how genes, environment, and lifestyle contribute to health and disease. These researchers would study all kinds of diseases.

Participants could have control over how their information is shared and used in research. They might get access to the information collected about their health.
