# Supplementary material for: A Survey of U.S Adults’ Opinions about Conduct of a Nationwide Precision Medicine Initiative® Cohort Study of Genes and Environment
Source: PLoS One. 2016 Aug 17;11(8):e0160461. doi: 10.1371/journal.pone.0160461 (PMC4988644; doi:10.1371/journal.pone.0160461)
Supplement: S2 Appendix — (DOCX) [file pone.0160461.s002.docx]

**S2 Appendix: Exact wording of survey questions used in this manuscript.**

Below are exact wordings for the questions analyzed in this paper.

Based on the description you just read, do you think this study should be done?

Definitely yes

Probably yes

Probably no

Definitely no

Based on the description you just read, would you participate in the study if you were asked? [Prompt once if skipped “We would really appreciate your answer to this question”]

Definitely yes

Probably yes

Probably no

Definitely no

When thinking about whether or not you would take part in this study, do you agree or disagree with the following statement?

Participating in the study would take too much of my time.

Strongly agree

Agree

Disagree

Strongly disagree

Now we’d like to ask your opinions about some parts of the study design.

If you joined the study, would you be willing to participate for:

1 year

5 years

10 years

20 years

My lifetime

I would not join the study

Imagine you were considering participating in the study. Would you be willing to provide:

a. A blood sample.

b. Other samples, like urine, saliva, or hair.

c. Your genetic information.

d. Your family’s medical history

e. Information about your lifestyle (diet, exercise, etc.)

f. Information collected from a mobile device (like a Fitbit or smartphone) about things like your heart rate or daily number of steps taken.

g. Information from your social media account(s).

h. Measures of the soil or water from your home.

[Yes]

[No]

Do you have a cell phone?

Yes

No

To the best of your knowledge, is the cellphone you use most often a smartphone?

Yes

No

If you were texted or prompted on your cell phone to answer a question from the study, or measure your pulse, how many times per day would you be willing to respond?

1. I would not be willing.
2. Once a week
3. Once a day
4. 2-3 times per day
5. 4-5 times per day
6. 6 or more times per da

Would you allow the following types of researchers to use your samples and information for research?

a. Researchers at the National Institutes of Health.

b. Other government researchers

c. University researchers in the United States

d. Pharmaceutical or drug company researchers

e. University researchers in other countries

Yes

No

In deciding whether or not to participate, how important would the following incentives be to you?

a. Having a free Internet connection during the study.

b. Learning information about my health.

c. Getting health care while participating in the study.

d. Receiving payment for my time.

e. Getting a free smartphone and data plan.

f. Getting a free activity tracker like a Fitbit, Jawbone, or Misfit.

Very important

Somewhat important

Not very important

Not important at all

Do you agree or disagree with the following statement?

Research participants and researchers should be equal partners in the study.

Strongly agree

Agree

Disagree

Strongly disagree

Do you agree or disagree with the following statements?

Research participants should:

a. help design the study

b. help choose what research questions to answer.

c. help decide what kinds of research are appropriate.

d. help recruit other participants.

e. help collect study data.

f. help analyze the data.

g. help decide what to do with study results.

Strongly agree

Agree

Disagree

Strongly disagree

Which aspects of the study would *you* want to be involved in?

1. Helping design the study.
2. Helping choose what research questions to answer.
3. Helping decide what kinds of research are appropriate.
4. Helping recruit other participants.
5. Helping collect study data.
6. Helping analyze the data.
7. Helping decide what to do with study results.

If research participants helped plan and run the study, would that change your willingness to participate?

1. I would be more willing to participate.
2. I would be less willing to participate.
3. It would not affect my willingness to participate.

If the study went forward, as a study participant what types of information would you like to receive?

a. A copy of my medical record.

b. My genetic results.

c. Health information based on my family history.

d. Ancestry information based on my genetics.

e. Lab results (cholesterol, blood sugar, etc.).

f. Nutritional information (body mass index, dietary analysis).

g. Information about your water or air quality.

h. How my health and lifestyle compare to other participants.

i. Information about other research studies related to my health.

(Following the consent description shown in Appendix 3 below)

Would you consent to share your samples and information with researchers in this

manner?

a. Definitely yes

b. Probably yes

c. Probably not

d. Definitely not

Do you agree or disagree with the following statement?

I trust the study to protect my privacy.

Strongly agree

Agree

Disagree

Strongly disagree

Do you agree or disagree with the following statement?

If my personal information (like name, address, and contact information) was removed first, I would be willing to have my information and research results available on the Internet to anyone.

a. Agree

b. Disagree

Now that you have had a chance to think about the study, would you participate in the study if you were asked?

Definitely yes

Probably yes

Probably no

Definitely no
