# Supplementary material for: A Survey of U.S Adults’ Opinions about Conduct of a Nationwide Precision Medicine Initiative® Cohort Study of Genes and Environment
Source: PLoS One. 2016 Aug 17;11(8):e0160461. doi: 10.1371/journal.pone.0160461 (PMC4988644; doi:10.1371/journal.pone.0160461)
Supplement: S3 Appendix — (DOCX) [file pone.0160461.s003.docx]

**S3 Appendix: Wording used to describe eight consent scenarios in the survey.**

Participants were randomized to see one of the following four descriptions of consent for the study. Additionally, half of participants saw the statement below, immediately following the consent description, while the other half did not: “You would (also) have access to a website where you would be able to see what studies are going on, which studies are using your information, and what each study has learned.”

BROAD/TRANSPARENT CONSENT

“Imagine you were asked to participate in the study. If you consented, researchers who get approval from the oversight committee would be allowed to use your samples and information for a broad range of research topics.”

STUDY-BY-STUDY

“Imagine you were asked to participate in the study.

If you consented, then each time a researcher got approval from the oversight committee to do a research project, you would be asked for your permission to use your samples and information for that project.”

MENU

“Imagine you were asked to participate in the study.

If you consented, at the beginning of the study you could select the kinds of research (for example, cancer, diabetes) that you would or would not want your samples and information used for.

Researchers who get approval from the oversight committee would be allowed to use your samples and information for the topics you choose.”

DYNAMIC

“Imagine you were asked to participate in the study.

If you consented, researchers who get approval from the oversight committee could use your samples and information for a broad range of research topics.

You would have access to a private, password protected website where you could decide not to be part of particular research studies, and decide what information to share and who to share it with.”
